# Research on fast-charging battery thermal management system based on refrigerant direct cooling

**DOI:** 10.1038/s41598-023-38330-3

**Published:** 2023-07-20

**Authors:** Naichang Dai, Jiangqi Long

**Affiliations:** 1 School of Intelligent Manufacturing, Wenzhou Polytechnic, Wenzhou, 32500 China; 2 School of Mechanical and Electrical Engineering, Wenzhou University, Wenzhou, 325035 China

**Keywords:** Engineering, Mechanical engineering

## Abstract

Aiming at the problem of high battery heat generation during the super fast-charging process of electric vehicle fast-charging power batteries, this study designs a fast-charging battery thermal management system based on the refrigerant direct cooling architecture. In order to use the refrigerant of refrigerant to cool the battery quickly. Firstly, the study constructs the heat generation model of the power battery, the calculation model of the battery thermal management system, and builds the experimental device. Secondly, theoretical simulations and experimental studies were conducted for low-temperature fast-charging and high-temperature fast-charging operating conditions. The experimental results show that the designed battery thermal management system has good cooling effect and temperature uniformity.

## Introduction

With the rapid development of new energy vehicle technology, the range of new energy vehicles is becoming a pain point for the majority of car owners. At present, two major solutions are available to try to solve the range problem of new energy vehicles. One is the battery super fast-charging technology, and the other is the battery quick change technology. From the current development, super fast-charging technology is the mainstream direction of technology to solve the problem of new energy vehicle range^1^.

The advantages of super fast-charging technology for power batteries are clearly evident. On the one hand, the charging speed is fast, and on the other hand, the charging efficiency is high. The emergence of super fast-charging technology for power batteries has provided great convenience for users of new energy vehicles. However, a series of problems can be caused by the emergence of super fast-charging technology for power batteries. The frequent use of excessive current charging will make the high-speed movement of lithium ions in the process of generating a large amount of heat, resulting in a sharp rise in battery temperature. Overheating of the battery makes the lithium ion in the charging process may be too late to complete the negative electrode embedding affect the cycle life of the battery, while the continuous overheating of the battery can also cause the risk of spontaneous combustion or explosion of lithium-ion batteries. Therefore, how to effectively control the temperature of the power battery during fast-charging is one of the important research contents in the development of thermal management system of power battery system^2^.

Refrigerant direct cooling technology is a new type of power battery phase change cooling system, which uses the refrigerant in automotive air conditioners as a cooling medium and introduces it into the evaporator of the power battery to achieve the purpose of rapid cooling of the power battery. Compared with other power battery cooling technologies, direct refrigerant cooling not only has higher cooling efficiency, but also can significantly reduce the cost of the whole vehicle, which is an important development direction for future power battery thermal management system design. In this paper, we will take the fast-charging power battery thermal management system with direct cooling as the research object, and provide useful exploration for the design of power battery thermal management system through simulation and experimental comparison analysis^3–5^.

## Heat generation characteristics of fast-charging power batteries

The actual heat production of lithium-ion batteries is complex, and the following simplified calculation model can be used in the simulation calculation.

1 $$\rho c_{p} \frac{\partial T}{{\partial t}} = \lambda_{x} \frac{{\partial^{2} T}}{{\partial x^{2} }} + \lambda_{xy} \frac{{\partial^{2} T}}{{\partial y^{2} }} + \lambda_{zx} \frac{{\partial^{2} T}}{{\partial z^{2} }} + q$$

where *T* refers to the temperature; *t* refers to the time; *ρ* means the average density of the material inside the Li-ion battery; *q* indicates the heat production rate per unit volume of the Li-ion battery; and *λ*_*x*_, *λ*_*y*_, *λ*_*zx*_ refers to the thermal conductivity of the Li-ion battery in the three-dimensional orthogonal direction. To solve the equations of the battery thermal effect model, it is necessary to obtain the thermal physical parameters of the battery, which contain the constant pressure specific heat capacity *c*_*p*_ and the thermal conductivity *λ*_*x*_, *λ*_*y*_, *λ*_*z*_ of the battery. where the thermal conductivity is calculated by theoretical estimation and finite element method.

The formula for calculating the constant voltage specific heat capacity of a lithium-ion battery is as follows.

2 $$c_{p} = \frac{1}{m}\sum\nolimits_{i}\left( {c_{i} \cdot m_{i} } \right)$$

where *m* denotes the mass of a single lithium-ion battery; *c*_*p*_ is the average constant pressure specific heat capacity of a single cell; *m*_*i*_ refers to the mass of each material inside the single cell; and *c*_*i*_ indicates the average specific heat capacity of each material inside the battery cell^6,7^. The thermal capacity of the battery pack is equal to the single cell thermal capacity multiplied by the number of individual cells.

When estimating the battery heat production rate, the following equation is used for the battery heat production rate model^6^.

3 $$q = \frac{I}{{V_{b} }}\left[ {(E_{0} - U_{1} ) - T\frac{{dE_{0} }}{dT}} \right]$$

where *V*_*b*_ refers to the volume of the battery cell; *I* means the current of charging and discharging the battery (A); *E*_0_ is the open circuit voltage of the battery; *U*_1_ indicates the terminal voltage of the battery; *T* means the thermodynamic temperature (K); $\frac{{dE_{0} }}{dT}$ refers to the temperature effect coefficient (V/K); (*E*_0_ − *U*_1_) means the Joule heat generated by the battery reaction; and $T\frac{{dE_{0} }}{dT}$ is the reversible reaction heat generated by the battery reaction.

$T\frac{{dE_{0} }}{dT}$ refers to the physical quantity associated with the electrochemical reaction and can be considered as a constant value for a given battery. We get $T\frac{{dE_{0} }}{dT} = 11.16\,{\text{mv}}$, $V_{b} = 0.00551088\,{\text{m}}^{3}$, and $R_{0} = 1.15\,{\text{m}}\Omega$ from the experimental data of the fast-charging battery. Finally, the expression of the heat production rate of a single cell is derived as follows.

4 $$q = \frac{I}{{V_{b} }}\left( {I^{2} R_{0} - T\frac{{dE_{0} }}{dT}} \right)$$

Normally, the battery pack heat dissipation is done by forced convection, which is divided into laminar flow and turbulent flow. It is also discriminated by Reynolds number *R*_*e*_, which is given by the following equation.

5 $$R_{e} = \rho LV_{0} /\mu$$

where *ρ* refers to the fluid density; *L* refers to the characteristic scale; *V*_0_ is the fluid flow velocity; and *μ* is the dynamic viscosity of the fluid. In the case of liquid-cooled convection heat dissipation, the *R*_*e*_ is generally greater than 2300 for turbulent flow and there is a local laminar flow situation. The standard two-equation *k*–*ε* model + Two-Layer ALL y + Wall Treatment model is usually used in STAR-CCM+^6–10^.

**Figure 1:**
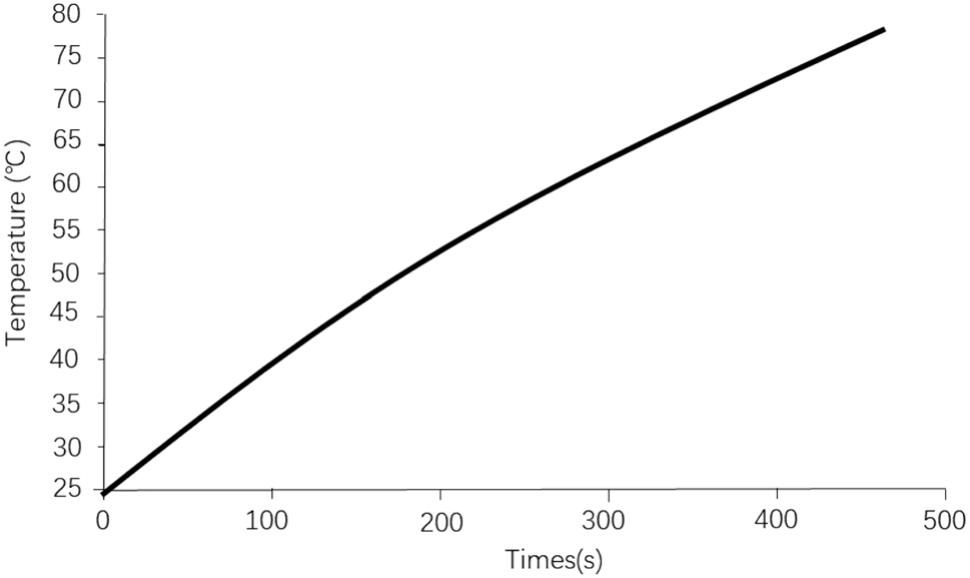
The battery temperature rises during the 6C fast-charging process.

Figure 1 shows the temperature rise of a fast-charging battery under constant current 6C charge at ambient temperature of 25 °C without cooling. The fast-charging single battery capacity is 95Ah, while the 6C multiplier fast-charging current is 570A. The test data in Fig. 1 shows that without cooling and high current fast-charging, the battery temperature will rise sharply. 480 s or so the battery temperature has exceeded 75 °C, the average temperature rise is > 0.1 °C/s, this temperature has far exceeded the upper protection threshold for normal use of the power battery. Without effective rapid cooling, the power battery will not be able to fast-charging at very high speed. In fact, when the temperature of the battery exceeds the upper protection threshold of the power battery (> 45 °C), the charging capacity of the battery drops sharply from the perspective of battery protection. Maintaining high current fast-charging will damage the power battery, which is not allowed. Therefore, if you want to continue to maintain the high-current fast-charging mode in the case of rapid fast-charging of the power battery, the temperature of the power battery must be strictly controlled below 45 °C, which is the basic goal of the power battery thermal management system design.

Table 1 gives the MAP table of the fast-charging battery obtained from the measurement. From the data in the table, we can see that after the battery temperature exceeds 45 °C, the charging capacity of the power battery drops sharply; beyond 50 °C, the charging multiplier drops to less than 0.5C. Therefore, when designing the battery thermal management system, it is necessary to fully consider the heat dissipation of the power battery in the fast-charging state. Only by always controlling the temperature of the battery within the appropriate operating range can the battery be maintained to charge with high efficiency, while ensuring the good safety of the power battery use.

**Table 1:** Charge map of fast-charging battery.

T/SOC	0%	10%	20%	30%	40%	50%	60%	70%	80%	90%	100%
− 20 °C											
Cut-off volt	3.150	3.639	3.706	3.743	3.775	3.815	3.874	3.965	4.075	4.120	4.200
Charge Rate	0.05C	0.10C	0.10C	0.10C	0.10C	0.10C	0.10C	0.07C	0.07C	0.07C	–
− 10 °C											
Cut-off volt	3.150	3.639	3.706	3.743	3.775	3.815	3.874	3.965	4.075	4.120	4.200
Charge Rate	0.05C	0.20C	0.20C	0.20C	0.20C	0.20C	0.20C	0.20C	0.20C	0.10C	–
0 °C											
Cut-off volt	3.150	3.639	3.706	3.743	3.775	3.815	3.874	3.965	4.075	4.120	4.200
Charge Rate	0.05C	0.33C	0.33C	0.33C	0.33C	0.33C	0.33C	0.33C	0.33C	0.20C	–
10 °C											
Cut-off volt	3.150	3.639	3.706	3.743	3.775	3.815	3.874	3.965	4.075	4.120	4.200
Charge Rate	0.05C	1.00C	1.00C	1.00C	1.00C	1.00C	1.00C	1.00C	1.00C	0.60C	–
20 °C											
Cut-off volt	3.150	3.639	3.706	3.743	3.775	3.815	3.874	3.965	4.075	4.120	4.200
Charge Rate	0.05C	3.00C	3.00C	3.00C	3.00C	3.00C	2.50C	2.50C	2.00C	1.00C	–
40 °C											
Cut-off volt	3.150	3.639	3.706	3.743	3.775	3.815	3.874	3.965	4.075	4.120	4.200
Charge Rate	0.05C	3.00C	3.00C	3.00C	3.00C	3.00C	3.00C	3.00C	3.00C	1.00C	–
45 °C											
Cut-off volt	3.150	3.639	3.706	3.743	3.775	3.815	3.874	3.965	4.075	4.120	4.200
Charge Rate	0.05C	1.00C	1.00C	1.00C	1.00C	1.00C	1.00C	1.00C	1.00C	0.50C	–
50 °C											
Cut-off volt	3.150	3.639	3.706	3.743	3.775	3.815	3.874	3.965	4.075	4.120	4.200
Charge Rate	0.05C	0.05C	0.05C	0.05C	0.05C	0.05C	0.05C	0.05C	0.33C	0.20C	–
55 °C											
Cut-off volt	3.150	3.639	3.706	3.743	3.775	3.815	3.874	3.965	4.075	4.120	4.200
Charge Rate	0.05C	0.33C	0.33C	0.33C	0.33C	0.33C	0.33C	0.33C	0.33C	0.20C	–

## Design of fast-charging power battery thermal management system based on direct cooling of refrigerant

**Figure 2:**
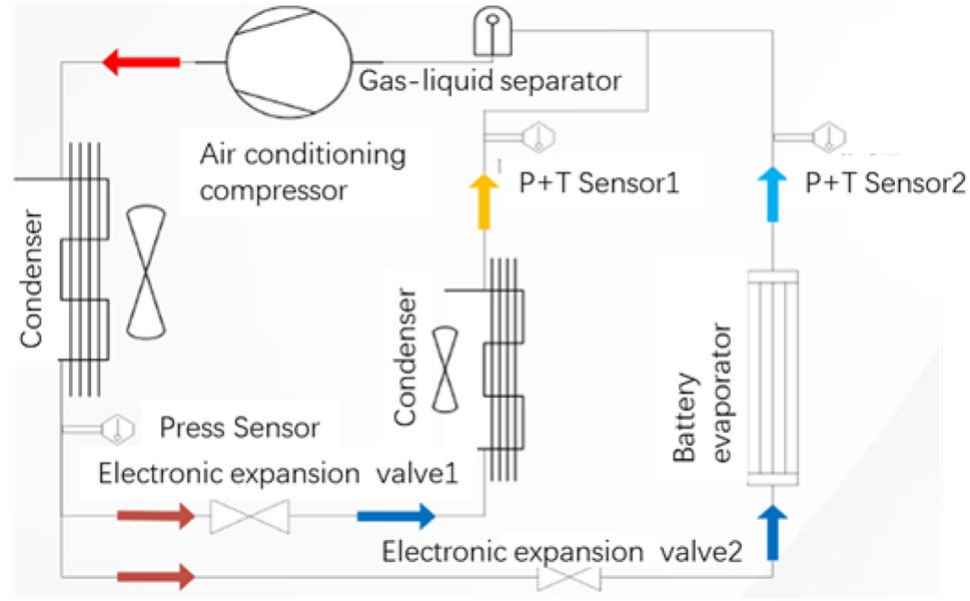
The refrigerant direct cooling parallel system diagram for power battery.

Due to the high cooling efficiency of direct refrigerant cooling, the direct refrigerant cooling technology is applied to achieve rapid cooling during the super fast-charging of power batteries. Figure 2 shows the diagram of the power battery direct cooling system. An electronic expansion valve and a P–T sensor are added to the evaporator circuit and the battery cooling circuit, respectively, since the power battery cooling system needs to work together with the air conditioning system to achieve the cooling function. Meanwhile, in order to avoid the problem of compressor liquid strike, a gas–liquid separator is added before the low pressure enters the compressor.

The heat exchange equation of the refrigerant exported from the air conditioning compressor in the condenser and evaporator is as below^11^.

6 $$\varphi_{in} = h_{in} \times A_{in} \times (T_{r} - T_{\omega } )$$

7 $$\varphi_{out} = h_{out} \times A_{out} \times (T_{a} - T_{\omega } )$$

where *h*_*in*_ and *h*_*out*_ denote the convective heat transfer coefficients at the inlet and outlet positions of the air conditioning compressor, respectively; *φ*_*in*_ and *φ*_*out*_ refer to the heat flow densities at the inlet and outlet positions of the air conditioning compressor, respectively; *A*_*in*_ and *A*_*out*_ indicate the heat transfer areas at the inlet and outlet positions of the air conditioning compressor, respectively; *T*_*r*_ is the temperature of the refrigerant; *T*_*a*_ is the gas temperature; and *T*_*ω*_ is the temperature of the pipe wall.

The heat transfer equation at the intersection of solid and fluid is as follows.

8 $$\lambda \left( {\frac{\partial T}{{\partial X}}} \right)_{W} = h\left( {T_{W} - T_{L} } \right)$$

9 $$q = q_{c} + q_{b}$$

10 $$q_{c} = h_{c} \left( {T_{W} - T_{1} } \right)$$

11 $$h_{c} = 0.023{\text{Re}}^{0.8} \Pr^{0.4} \left( {\lambda /D} \right)$$

12 $$q_{b} = \mu r\sqrt {\frac{{g\left( {\rho_{1} - \rho_{v} } \right)}}{\sigma }} \left( {\frac{{C_{p1} \left( {T_{W} - T_{sat} } \right)}}{{C_{q} r\Pr^{n} }}} \right)$$

where *λ* is the average thermal conductivity of gas–liquid volume; *q* indicates the total heat transfer; *q*_*c*_ denotes convection enhanced heat transfer; *h*_*c*_ refers to the convection heat transfer coefficient; *q*_*b*_ means boiling heat transfer; *C*_*p*1_ is the specific heat capacity of liquid phase; *r* represents the latent heat of phase change; *σ* denotes the surface tension coefficient of liquid phase. Equations (8–12) will be used as the basis for the calculation of the simulation model later^12^.

The internal resistance of battery is a Strongly correlated material function with the depth of battery charge and discharge. With the increase of the depth of discharge, the total internal resistance increases regularly. The total internal resistance of the battery will constantly change with the charging and discharging process, because the activity of the positive and negative electrode materials inside the battery, the concentration, density, and temperature of the battery electrolyte are all constantly changing in conjunction with the charging and discharging conditions. According to the electrochemical theory, the ohmic internal resistance of the battery obeys Ohm's law, and the polarization internal resistance of the battery is related to the current density inside the battery.

During the battery charging and discharging process, it is difficult to measure the polarization internal resistance of the battery using experimental tools because it is strongly related to factors such as the discharge depth and charging and discharging conditions of the battery, making it difficult to monitor in real-time. Therefore, this project adopts another processing method, which is based on the general model of battery heat generation established by Bernardi et al. The principle of this method is to treat the polarization heat and reaction heat together as irreversible reaction heat. Therefore, the heat generated during battery charging and discharging can be further simplified as:

13 $$Q_{t} = Q_{r}^{{\prime }} + I^{2} R_{e} + Q_{s}$$

$Q_{r}^{{\prime }}$ is the reaction heat including polarization heat.

Depending on the charging capacity shown in Table 1 for this fast-charging battery, the temperature setting of the power battery thermal management system is set from 20 °C to 45 °C. It is a temperature range for the best performance of the power battery and the best fast-charging capability, and all temperature control system design and control results are designed with this in mind^13^.

## Power battery thermal management system simulation calculation

The initial ambient temperature and battery temperature for the whole system simulation and experiment are set to 25 °C. Meanwhile, in order to ensure the cooling capacity of the air conditioning compressor, the temperature from the air conditioning compressor to the battery cooling inlet needs to be controlled to 15 °C under the ultra-high power fast-charging condition.

**Figure 3:**
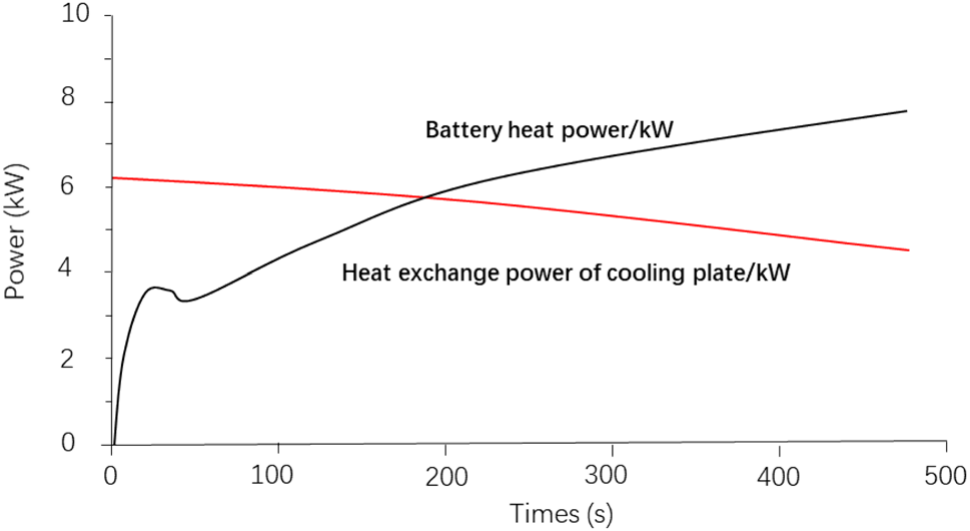
The relationship between the heating power of battery and the heat transfer power of water cooling plate.

The battery system parameters are shown in Table 2. The graphs of battery heating power and water-cooled plate heat exchange work variation were calculated without changing the parameters in the table (as shown in Fig. 3). Among them, the original vehicle is equipped with 34 cc displacement electric air conditioner; the initial ambient temperature and battery temperature are 25 °C; the initial SOC of the battery is 0. It can be seen from the simulation calculation results that the heating power of the power battery continues to increase with the extension of the high-power fast-charging time. In about 3 min, the heating power of the power battery has increased to about 6 kW. Thus, the heat transfer power of the battery cooling plate continues to decrease with the extension of the charging time. The heat exchange power of the battery cooling plate is already less than 6 kW when charging to 3 min. With the extension of charging time, the heat transfer power of the cooling plate can not meet the heating power of the battery. This means that as the charging process continues, the battery temperature will continue to rise, and the purpose of controlling the battery temperature cannot be achieved.

**Table 2:** Battery system parameter table.

Test project	Battery capacity (Ah)	Total energy (kWh)	Nominal voltage/maximum voltage (V)	Peak charging current (A)
Performance parameters	95	73.8	770/894	570

With the existing model parameters, the cell temperature parameters can be calculated for different liquid-cooled plate inlet temperatures and flow rates, and the results are shown in Table 3. From the data in Table 3, it can be seen that: (1) when fast-charging for 8 min makes SOC charging from 0 to 80%, under the flow rate of 20L/min of battery cooling system, even if the liquid cooling plate inlet coolant temperature is reduced to 10 °C, the maximum temperature of the battery still exceeds the control target of 45 °C, which cannot meet the cooling demand of the power battery in the process of high power fast-charging. And in reality, it is difficult to control the temperature of the electric air conditioning compressor outlet of electric vehicles to 10 °C. Therefore, the control target of 10 °C for the air conditioner compressor outlet temperature is not realistic and feasible. (2) When the flow rate of the battery cooling system is 10L/min, it cannot meet the heat exchange target of the power battery. Therefore, the flow rate of the battery cooling system needs to be increased to 20 L/min. In the case that the temperature of the outlet of the air conditioning compressor is controlled to 15 °C and the flow rate of the battery cooling system is increased to 20 L/min, it is still not possible to control the temperature of the power battery. Therefore, it is necessary to consider redesigning the parameters of the matching battery thermal management system.

**Table 3:** Simulation results of battery cooling system.

Liquid cooling plate inlet temperature and flow rate	Maximum battery temperature (°C)	Maximum battery temperature difference (°C)	Maximum heat exchange of cold plate (kW)
10 °C@10L/min	55.0	4.5	10.6
15 °C@10L/min	56.0	4.2	9.7
20 °C@10L/min	56.8	4.0	8.3
10 °C@20L/min	54.2	4.6	11.3
15 °C@20L/min	56.0	4.2	9.6
20 °C@20L/min	56.6	3.9	9.2

### Results of air conditioning compressor performance simulation analysis

**Figure 4:**
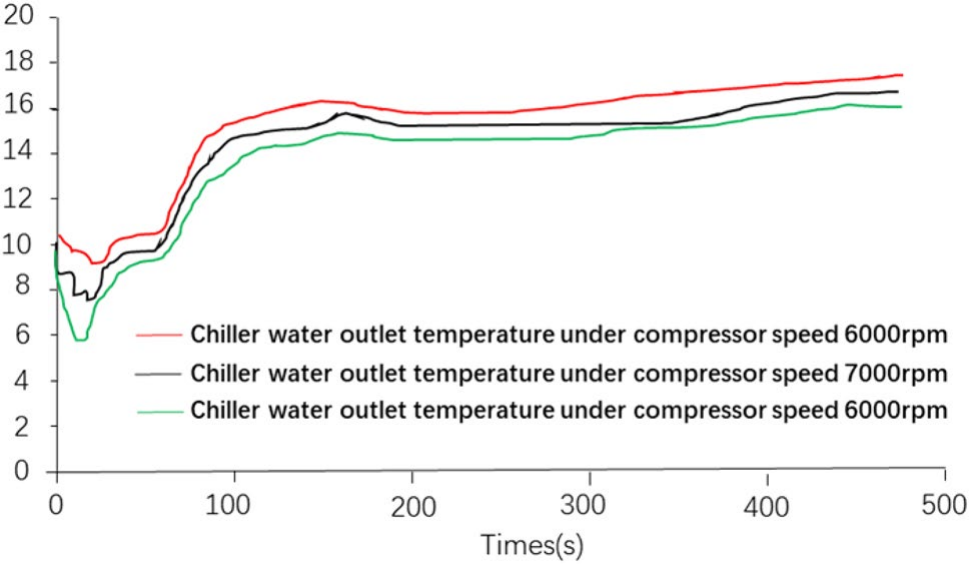
Cooling temperature of air conditioner compressor outlet at different discharge rate and rotational speed.

Figure 4 shows the relationship between air conditioning compressor displacement, rotational speed and outlet cooling temperature obtained from the simulation calculation. From the diagram, it can be seen that for the 34 cc air conditioning compressor, even at the speed of 8000 rpm, the control temperature at the outlet of the air conditioning compressor is still around 17 °C, which is not up to the design requirement of 15 °C control temperature. The temperature at the outlet of the air conditioner compressor can be controlled at about 15 °C when the 56 cc air conditioner compressor is selected, after the speed reaches 7000 rpm and the power of the water pump of the cooling circuit is increased. When the speed reaches 8000 rpm, the temperature at the outlet of the air conditioner compressor can be stabilized at about 14 °C.

**Figure 5:**
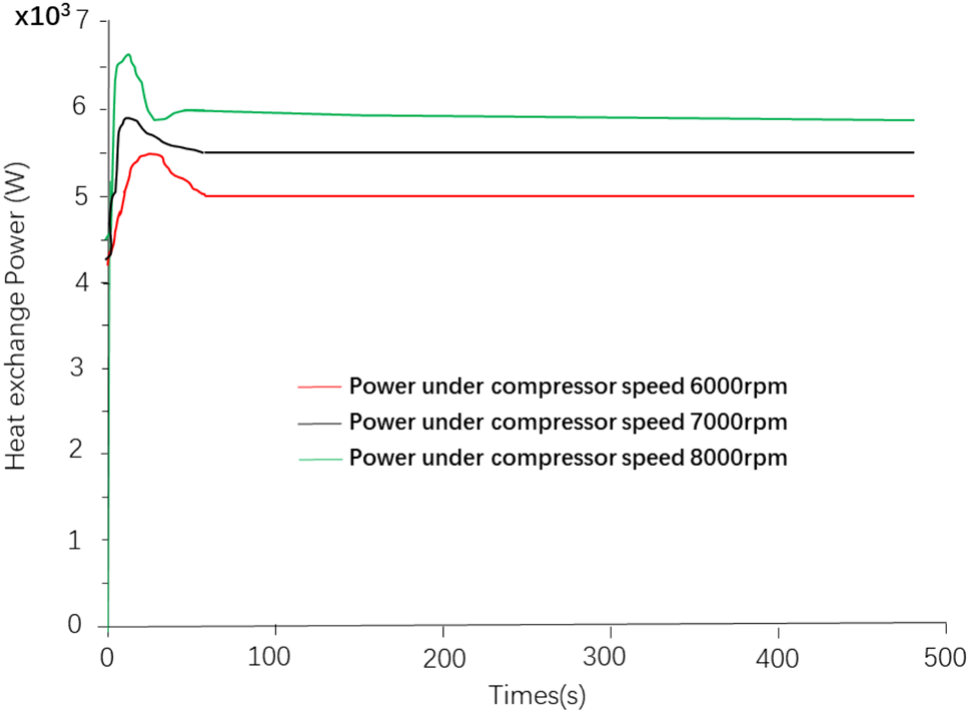
Battery cooling board Heat transfer power at different rotational speeds.

The heat transfer power of the battery cooling plate veneer of the 56 cc air conditioning compressor at different rotational speeds is shown in Fig. 5. It can be seen from the figure that the heat transfer power of the battery cooling plate veneer can be stabilized to about 6 kW at 8000 rpm. Even at 6000 rpm, the heat transfer power of the cooling plate veneer can be stabilized at about 5 kW.

### Results of cooling performance simulation analysis of battery cooling system

**Figure 6:**
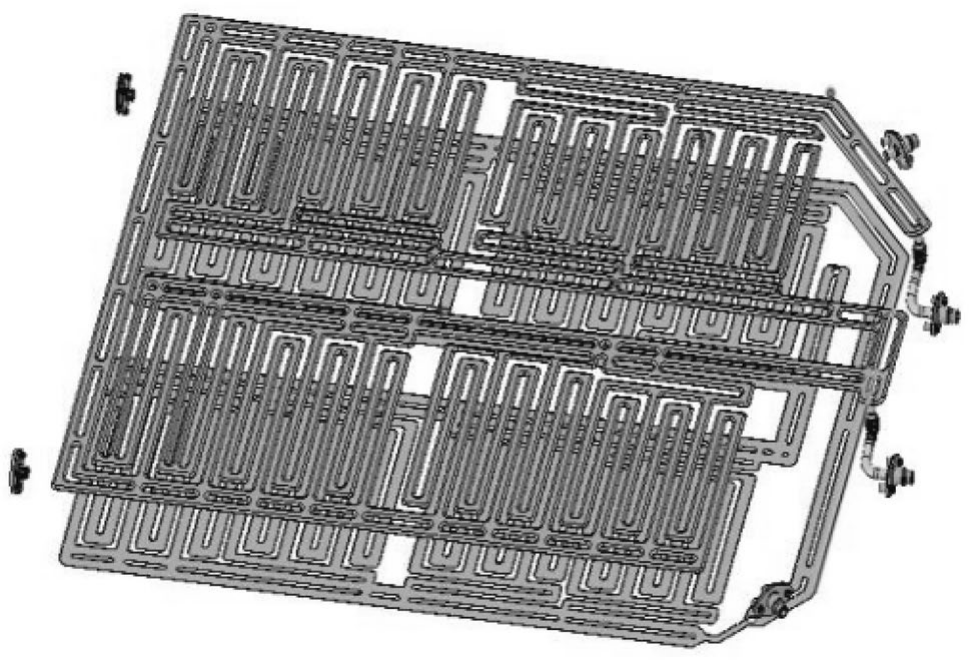
Double layer cooling plate scheme for battery module.

From the previous simulation analysis results, it can be seen that the power cell temperature exceeds 54 °C under large multiplier fast-charging conditions, and the heat transfer power of the battery cooling single plate cannot meet the heat dissipation demand of the power cell. Therefore, in the design of the cooling structure of the battery system, it is necessary to consider the use of dual cooling plate cooling mode. In other words, in addition to the cooling plate at the bottom of the battery pack, a set of liquid cooling plate is also added to the upper part of the power battery module, forming a double liquid cooling plate structure (as shown in Fig. 6). The design parameters of the liquid cooling plate are the same as the design parameters of the liquid cooling plate at the bottom of the battery pack. Following this new design scheme, the simulation calculation model is modified and re-imported into the model for calculation and analysis.

Faced with the situation that the heat exchange of single layer liquid cooling plate is not enough to meet the heat dissipation demand of large rate charging of power battery. The new design solution maximizes the heat transfer capacity of the liquid cooling plate by increasing the heat transfer area of the liquid cooling plate. The subsequent simulation model is also modified to double the upper limit of the heat transfer capacity of the liquid cooling plate and recalculate it.

Table 4 gives the calculation results after optimization. As can be seen from the table, after increasing the cooling plate heat dissipation, upgrading the coolant flow to 20L/min and controlling the cooling temperature of the air conditioning compressor outlet to 15 °C, the battery can be controlled to the control target of the maximum battery temperature not exceeding 45 °C during the fast-charging process.

**Table 4:** Optimization simulation results of battery cooling system.

Initial ambient temperature/battery temperature (°C)	Maximum battery temperature (°C)	Maximum battery temperature difference (°C)	Maximum heat exchange of cold plate (kW)
25/25	42.2	2.5	6.4
30/30	42.9	2.5	7.5
35/35	43.8	2.6	8.3
40/40	45.0	2.8	9.6

**Figure 7:**
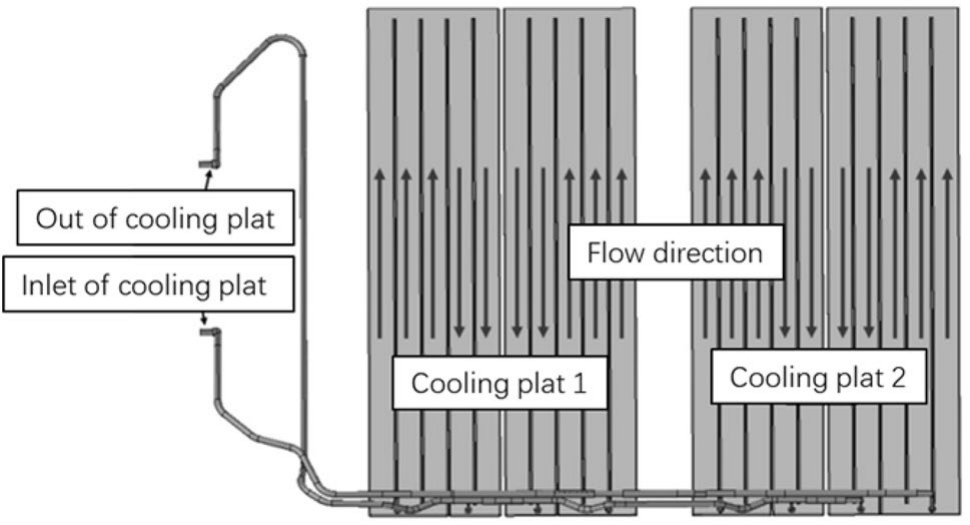
Design scheme of double layer cooling plate.

**Figure 8:**
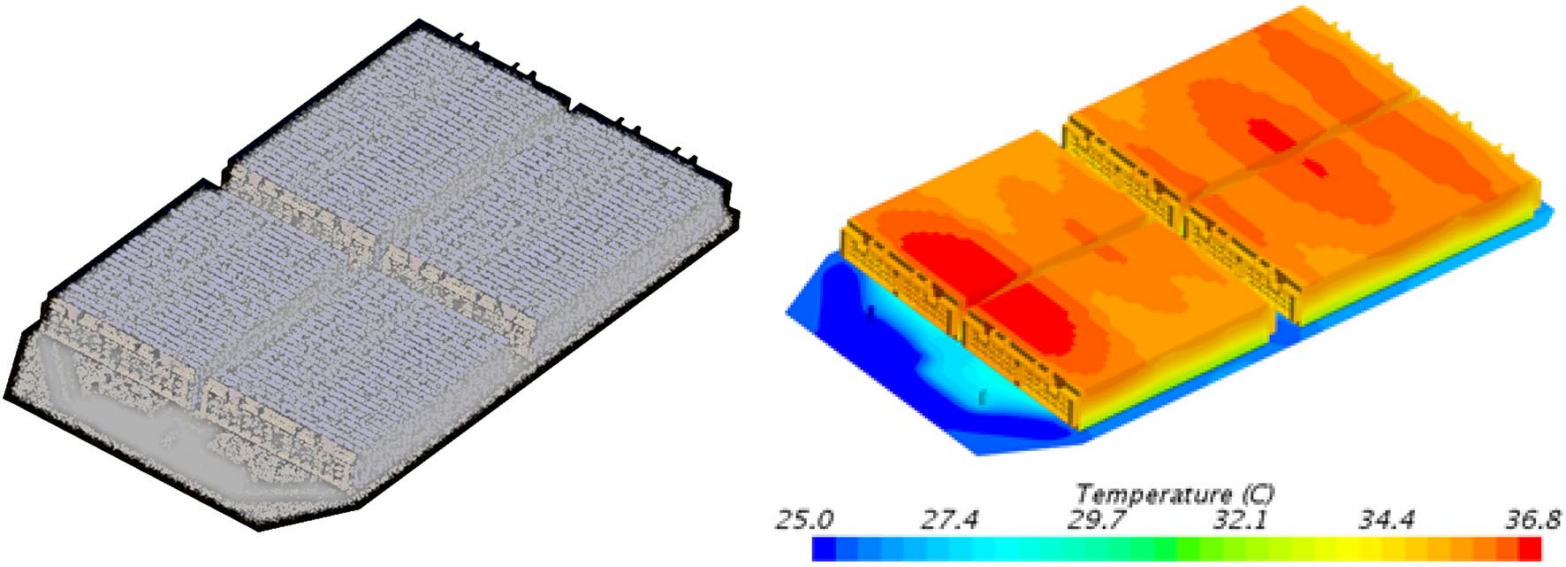
Geometric simulation model and Cloud chart of temperature distribution in battery pack under fast-charging condition.

According to the calculated results, the design parameters of the cooling plate are re-modified, as shown in Fig. 7. The pipe width of the double-layer cooling plate was modified to 18 mm, and the flow path direction was changed to transverse cooling structure. The simulation calculation was carried out for the power battery 6C fast-charging condition under the ambient temperature of 25 °C, and the simulation calculation results shown in Fig. 8 were obtained.

As shown by the simulation results, after adopting the double-layer liquid cooling scheme, the temperature of the power battery is well controlled under the normal temperature 6C multiplier fast-charging condition, which can be controlled below 36.8 °C, and the maximum temperature difference of the battery is 9.2 °C, which meets the design requirements.

**Figure 9:**
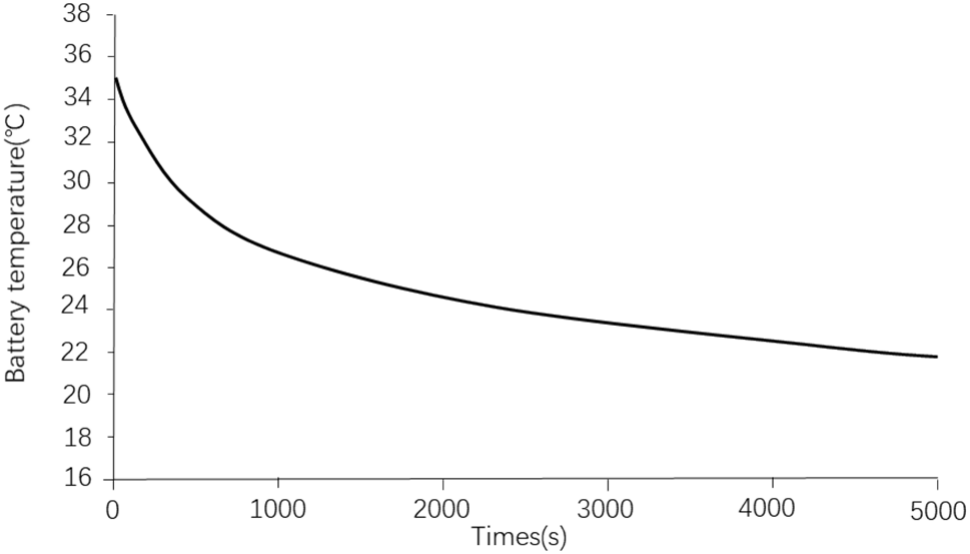
Battery temperature drop curve.

Figure 9 shows a diagram of the battery cooling process. The relevant parameters are set as follows: the power battery is in a high temperature environment of 40 °C; the light is 1060 W/m^2; the initial temperature of the passenger compartment is 50 °C; the temperature of the air conditioner condenser inlet is 43 °C; the inlet air speed is 3 m/s; the vehicle speed is 120 km/h; the initial temperature of the battery is 35 °C; the coolant flow rate is 20 L/min; and the air conditioner temperature is 25 °C. It can be seen from Fig. 9 that under the high speed condition of 120 km/h, the time to reduce the battery temperature from 35 °C to 25 °C is about 30 min, and the battery temperature reduction rate is 0.33 °C/min. This battery cooling time can be used as the battery pre-cooling time setting in the design of battery thermal management system strategy under the high temperature fast-charging condition of power battery.

**Figure 10:**
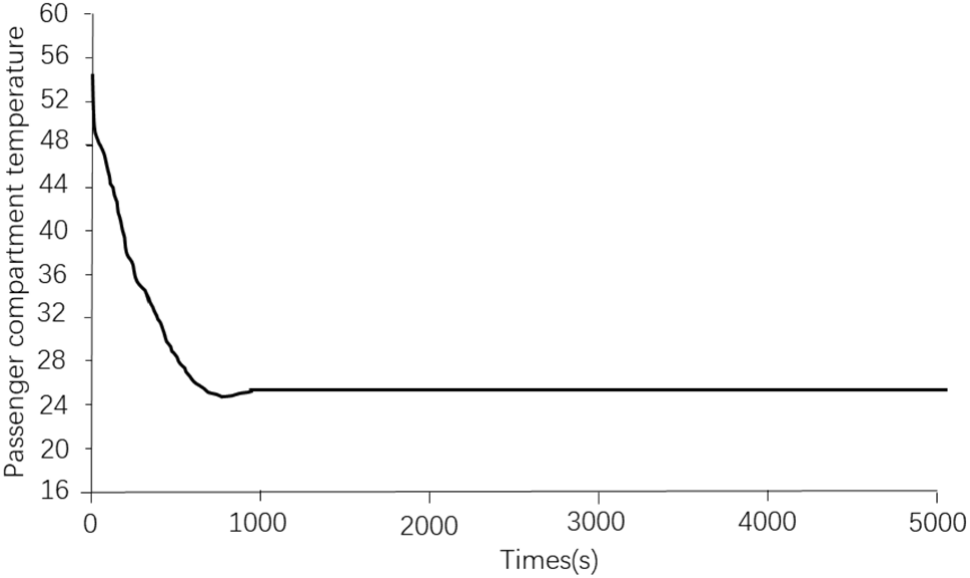
Crew cabin temperature control effect.

Figure 10 shows the temperature control effect of the electric vehicle passenger compartment under the corresponding working conditions. As can be seen from Fig. 10, the temperature of the passenger compartment of the EV can be reduced from 50 °C to the target control temperature of 25 °C after about 13 min. This result shows that the selected air conditioning displacement and cooling power can meet the needs of both battery cooling and occupant compartment cooling.

## Results of the experiment

**Figure 11:**
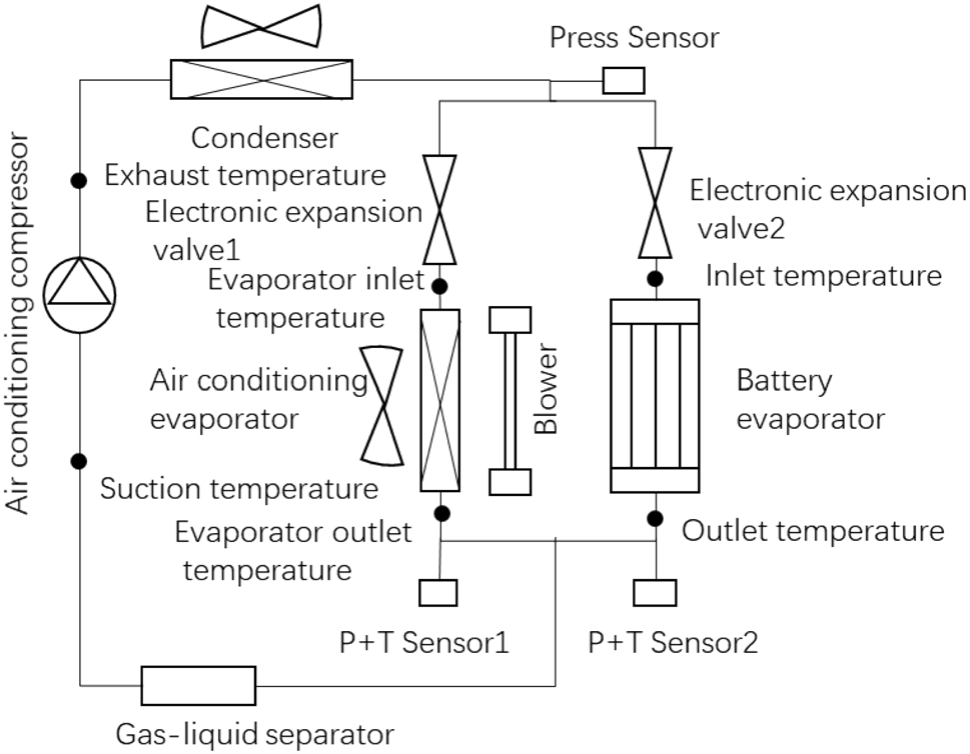
Experimental schematic diagram.

According to the results of the simulation analysis above, the control thresholds of the battery thermal management system and the performance parameters of the components were redesigned. The electric air conditioner compressor of 56 cc displacement was selected to replace the previous electric air conditioner of 34 cc displacement; the cooling fan of the cooling circuit was increased from 450 to 600W; the air conditioner condenser inlet speed was increased from 1.5 m/s to 2.2 m/s; the outlet control temperature was set to 15 °C; the air conditioner high pressure steady state value was 20 bar; the maximum speed of the air conditioner compressor was 8000 rpm The experimental schematic diagram of this paper is shown in Fig. 11. The experimental conditions are carried out according to the following three most representative extreme conditions.

The control objectives of the designed power cell system are as follows.

1. Total charging time < 2 h and difference between minimum and maximum battery temperature < 12 °C under fast-charging at − 20 °C.
2. The SOC should meet the 6C charging requirements (fast-charging time should be close to 6 min) during the period from 30 to 80% in the case of low temperature fast-charging at − 20 °C.
3. Total charging time < 1 h and SOC from 30 to 80% during fast-charging at room temperature 25 °C to meet 6C charging requirements (fast-charging time should be close to 6 min).
4. We need to start the air conditioning refrigerant direct cooling for battery cooling in the case of high temperature 40 °C fast-charging first. Total charging time < 1 h, and SOC from 30 to 80% period to meet the 6C charging requirements (fast-charging time to be close to 6 min).
5. Ambient temperature of 45 °C, the whole vehicle running high speed and overtaking working conditions power battery temperature > 45 °C.

Further, the battery thermal management system is verified to meet the design requirements according to the system control objectives.

### −20 °C low temperature high current fast-charging

**Figure 12:**
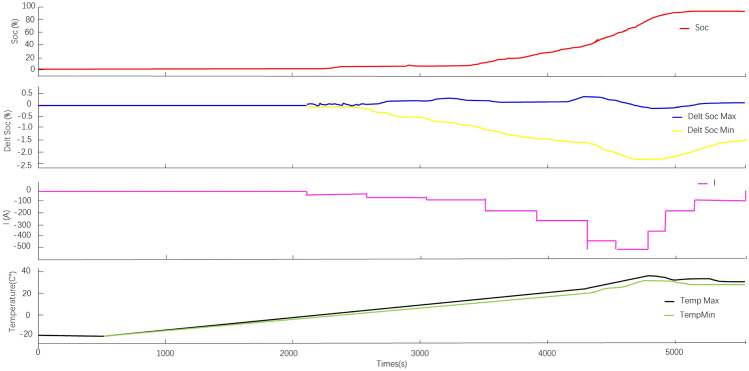
The fast-charging results under − 20 °C low temperature.

The refrigerant direct-cooled fast-charging battery pack is installed on the vehicle with initial SOC = 0, and the whole vehicle is mounted for low-temperature fast-charging test, and the − 20 °C low-temperature fast-charging results can be obtained (as shown in Fig. 12).

As can be seen from Fig. 12, the ambient temperature and battery temperature are relatively low in the low temperature environment. The lowest battery temperature is − 19.5 °C; the highest temperature is − 19.0 °C; the total charging time is 1h30min; the highest battery temperature during low temperature fast-charging is 35.6 °C; the lowest temperature is 31.5 °C; the maximum temperature difference is 4.1 °C; the charging current during SOC from 30 to 80% is 570A; the fast-charging time is about 6.3 min; the battery temperature during fast-charging < 45 °C.the experimental results meet the design requirements.

### High current fast-charging at 40 °C

**Figure 13:**
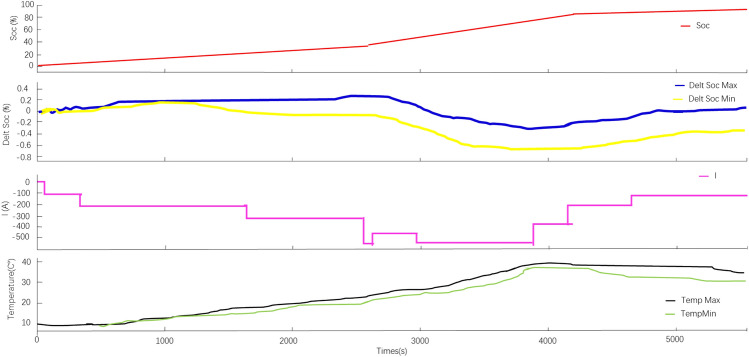
The fast-charging results under 40 °C low temperature.

The experimental data of fast-charging of the whole vehicle under high ambient temperature of 40 °C is shown in Fig. 13. The experimental data shows that the initial minimum temperature of the battery is 39.5 °C; the maximum temperature is 40.0 °C; the total charging time is about 40 min; the maximum temperature of the battery during high temperature fast-charging is 38.6 °C; the minimum temperature is 35.4 °C; the maximum temperature difference is 3.2 °C; the charging current during SOC from 30 to 80% is 570A; the fast-charging time is about 5.8 min; the fast-charging The battery temperature during the process < 45 °C. The experimental results meet the design requirements.

### 45 °C high speed and overtaking working condition test

**Figure 14:**
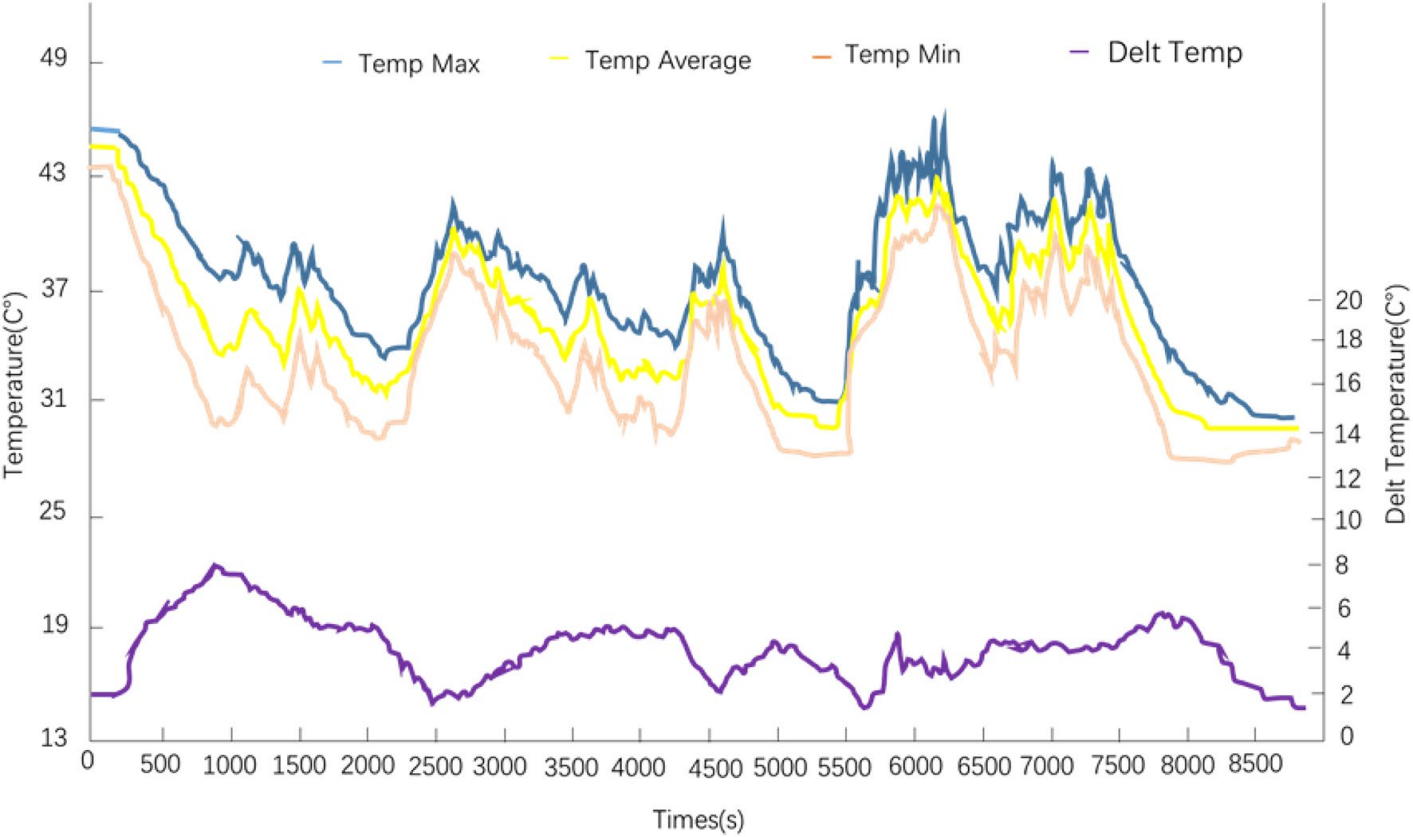
Experimental results of high speed and overtaking conditions at 45 °C.

The experimental results under the ambient temperature of 45 °C, the vehicle was subjected to high speed working condition and overtaking working condition are shown in Fig. 14. The maximum temperature of the initial battery is set to 44.0 °C and the minimum temperature is 43.3 °C in the experiment. From the data in Fig. 14, it can be seen that the maximum temperature of the power battery under high-speed driving and overtaking conditions < 44 °C. This indicates that the designed battery refrigerant direct cooling system can reduce the maximum battery temperature from 44 °C to 36 °C in a very short period of time. In the later high-speed overtaking conditions, the maximum temperature of the power battery < 44 °C; the maximum temperature difference is 6.2 °C. The experimental results meet the design requirements.

## Conclusion

In this paper, a series of analyses and studies are conducted on the thermal management system of fast-charging power battery with direct refrigerant cooling architecture. A simulation model is constructed based on the characteristics of direct refrigerant cooling and the charging characteristics of the fast-charging power battery, and the design parameters are imported into the simulation model for analysis and calculation. According to the results of the simulation calculation, the structure and design parameters of the thermal management system of the whole vehicle are re-matched and calculated, resulting in a new set of battery thermal management system adapted to the use of fast-charging power batteries.

The results of the research in this paper are as below:

1. The simulation calculation finds that the air conditioning displacement of the original vehicle cannot meet the heat exchange demand of the fast-charging battery in the fast-charging process, so the air conditioning liquid cooling system is re-selected and designed. Thus, the newly designed refrigerant direct cooling system can meet both the passenger compartment cooling requirements and the fast-charging heat exchange requirements of the fast-charging power battery.
2. Due to the fact that the single liquid cooling plate of the power battery cannot meet the heat exchange demand of the power battery, the study redesigned the double-layer liquid cooling plate scheme for the fast-charging battery. Both simulation and experimental results prove that the double-layer liquid-cooled plate solution can meet the heat exchange requirements of the fast-charging battery in low and high temperature environments, and the maximum temperature difference of the battery meets the design requirements.
3. In the study, experiments were conducted on the most typical low temperature charging, high temperature charging and high temperature and high speed overtaking conditions of electric vehicles, and the test results show that the designed battery thermal management system has good heat exchange effect.

The content and results of the study can provide useful references and lessons for the design of thermal management system for large multiplier fast-charging power batteries. In addition, the methods and results of the study are also useful for the design of thermal management systems of other types of power batteries.

## Data Availability

All datasets used and/or analyses carried out and results obtained are available from the corresponding author on reasonable request.

## References

[1] Zhu XQ, Wang ZP, Wang H (2020). Review of thermal runaway and safety management for lithium-ion traction batteries in electric vehicles. J. Mech. Eng..

[2] Jiang JC, Gao Y, Zhang CP (2019). Online diagnostic method for health status of lithium-ion battery in electric vehicle. J. Mech. Eng..

[3] Fu XF, Wang ML, Lai JJ (2021). Research on anti-electromagnetic interference ability performance of battery management system. Chin. J. Power Sources.

[4] Fu XF, Li G, Zeng WQ (2018). A research on power battery pack anti-vibration safety performance. Automob. Technol..

[5] Long JQ, Lan FC, Chen JQ (2008). New technology of lightweight and steel-aluminum hybrid structure car body. J. Mech. Eng..

[6] Liang JL (2020). The electrochemical and thermal performance of lithium batteries and an ultra-thin heat pipe-based thermal management system. South China Univ. Technol..

[7] Tomaszewska A (2019). Lithium-ion battery fast-charging: A review. Etransportation.

[8] Jochem P, Szimba E, Euter-oppermann M (2019). How many fast-charging stations do we need along European highways?. Transp. Res. D Transp. Environ..

[9] Chung Y, Kim MS (2019). Thermal analysis and pack level design of battery thermal management system with liquid cooling for electric vehicles. Energy Convers. Manag..

[10] Wang Y, Gao Q, Wang G (2018). A review on research status and key technologies of battery thermal management and its enhanced safety. Int. J. Energy Res..

[11] Fu XF, Zhou SJ, Zhai YX (2014). Analysis on the influence of battery management system CAN message asynchronous for PHEV control strategy and safety performance. J. Mech. Eng..

[12] Su ZG, Long JQ, Zhou SJ (2013). Safety simulation of rear-end collision and experimental study for extended range electric vehicle. China Mech. Eng..

[13] Cao, L. *Temperature Control and Optimization of Battery Thermal Management in Electric Vehicle* (Beijing University of Technology, 2019). 10.26935/d.cnki.gbjgu.2019.000416

